# *BRAF*‐mutated malignant melanoma with chondrosarcomatous differentiation in inguinal nodal metastasis

**DOI:** 10.1002/ccr3.3982

**Published:** 2021-03-07

**Authors:** Francesca Abbati, Annalisa Altimari, Barbara Corti, Emi Dika, Francesca Sperandi, Barbara Melotti

**Affiliations:** ^1^ Department of Medical Oncology IRCCS Azienda Ospedaliero‐Universitaria di Bologna Bologna Italy; ^2^ Laboratory of Oncologic Molecular Pathology IRCCS Azienda Ospedaliero‐Universitaria di Bologna Bologna Italy; ^3^ Pathology Unit Department of Diagnostic Medicine and Prevention Sant'Orsola‐Malpighi Hospital University of Bologna Italy; ^4^ Dermatology Department of Experimental, Diagnostic and Specialty Medicine University of Bologna Italy; ^5^ Department of Medical Oncology IRCCS Azienda Ospedaliero‐Universitaria Sant'Orsola ‐Malpighi Hospital Bologna Italy

**Keywords:** *BRAF* mutation, chondrosarcomatous differentiation, melanoma

## Abstract

We report the case of a young woman who developed metastatic melanoma in the inguinal nodal region, which acquired chondrosarcomatous differentiation and preserved the BRAF mutation found in the primary tumor. The patient was treated with a BRAF/MEK inhibitor combination therapy (dabrafenib/trametinib), which was demonstrated to be effective and well‐tolerated.

## Graphical abstract

BRAF‐mutated melanoma with chondrosarcomatoid differentiation and response to targeted therapy.

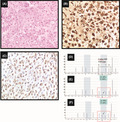

## 1 INTRODUCTION

Malignant melanoma is characterized by the development of significant morphological differences and atypical histopathological patterns vs normal cells. Chondrosarcomatous‐differentiated melanoma is extremely rare. We report the case of a young woman who developed metastatic melanoma in the inguinal nodal region, which acquired chondrosarcomatous differentiation and preserved the *BRAF* mutation found in the primary tumor. The patient was treated with a BRAF/MEK inhibitor combination therapy (dabrafenib/trametinib), which was demonstrated to be effective and well‐tolerated. To the best of our knowledge, this is the first case described in the literature of a metastatic recurrence of a melanoma that underwent a chondrosarcomatous differentiation with the same *BRAF* mutation (V600E) detected in the primary tumor. This case shows the importance of a correct clinical assessment in the diagnosis of melanomas, which are notable for their significant morphologic variability, and highlights the usefulness of immunohistochemical analysis in obtaining a definitive diagnosis. The good patient response to dabrafenib/trametinib combination therapy suggests that, in the rare chondrosarcomatous melanoma variant, *BRAF* mutation may be a predictive factor of response.

In addition to the ten different melanoma categories recognized by the WHO classification of tumors,^1^ many other histological variants have been described (recently reviewed ^2^, ^3^). Accurate diagnosis and classification of these other variants is important for improving patient management. Furthermore, the more uncommon histological variants of melanoma are known to display specific architectural patterns, stromal modifications, and cytological characteristics.^2^

Divergent differentiation of melanoma is a rare phenomenon, and when it occurs, it can be missed by unwary pathologists, consequently leading to diagnostic uncertainty.^4^ Currently, diagnosis of these melanomas relies on analysis of the size and shape of the tumor cells as well as identification of key cytoplasmatic, nuclear, architectural, and stromal characteristics. Obtaining a differential diagnosis between these rare histopathological melanoma variants and other soft tissue malignancies presents a significant clinical challenge.^5^, ^6^, ^7^

Chondrosarcomatous is an unusual histopathological variant of melanoma in which osteoid and chondroid metaplasia (ranging from purely osteoid/chondroid differentiation to a mixture of both) is detected in either the primary or the metastatic lesion.^3^ Melanomas with chondrosarcomatous differentiation are more common in acral locations, such as the subungual areas of toes and fingers, although cases with mucosal (nose, mouth, and vagina) and cutaneous onset (face, back, shoulder, ankle) have also been described.^8^

In this report, we describe an uncommon case of a relapsing melanoma that acquired the chondrosarcomatous phenotype and preserved some of the melanocytic markers and the *BRAF* mutation identified in the original tumor.

## 2 PATIENT PRESENTATION AND RESULTS

A 36‐year‐old woman underwent surgical resection of a cutaneous lesion situated on the abdominal wall. She had a good performance status and was not taking any chronic medications, and her personal and family history were negative for skin pathology.

**FIGURE 1:**
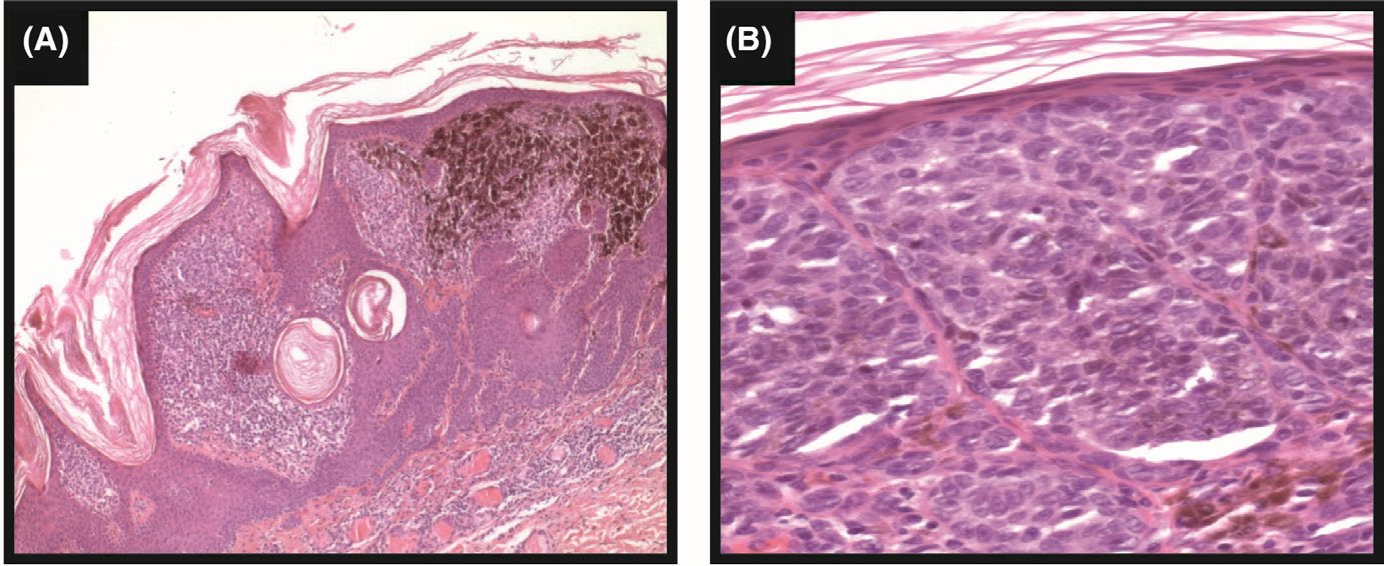
Histopathology of the primary cutaneous melanoma (hematoxylin‐eosin; original magnification 5× (A), and 20× (B))

Microscopic examination of the excised lesion revealed cutaneous melanoma with a Breslow thickness of 0.4 mm, <1 mitotic figure/mm^2^, no evidence of ulceration, regression, and vascular invasion, and negative surgical margins (Figure 1A and B).

Following clinical control identified the presence of a lymph adenomegaly in the right groin.

**FIGURE 2:**
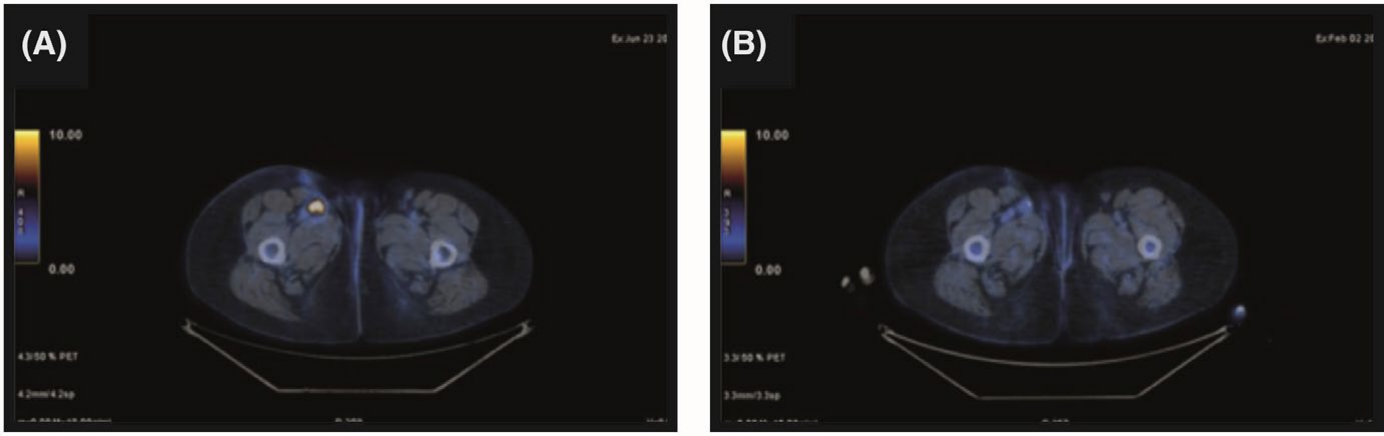
18F‐deoxyglucose positron emission tomography (FDG‐PET) conducted pretherapy demonstrating uptake in the right inguinal nodes (A) and after 7 mo of therapy, demonstrating substantial metabolic normalization in the inguinal nodes (B)

Investigation by 18F‐deoxyglucose positron emission tomography (FDG‐PET) showed the appearance of, at least, three inguinal pathological lymph nodes (Figure 2A). The two largest of these lymph nodes were 30 mm and 23 mm in size, respectively, with a maximum standardized uptake value (SUVmax) of 7 and 13, respectively.

After a multidisciplinary discussion of the case, patient underwent to a wide cutaneous excision at the primary lesion site along with complete right lymphadenectomy.

**FIGURE 3:**
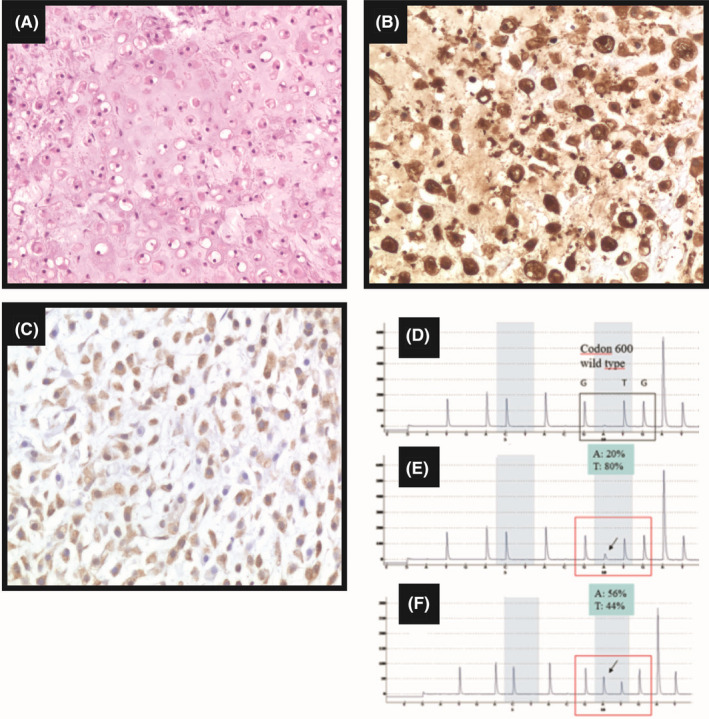
Histology of the nodal metastasis (A) (hematoxylin‐eosin; magnification 20×). Immunostaining for S‐100 (B) and HMB‐45 (C) (original magnification 20×). BRAF pyrogram: codon 600 wild type (D). BRAF pyrogram in the primary tumor: p.V600E (c.1799T > A), mutation frequency 20% (E); BRAF pyrogram in nodal metastasis: p.V600E (c.1799T > A), mutation frequency 56% (F)

Histological examination revealed nonresidual cutaneous neoplasia. The lymph node evaluation confirmed the diagnosis of metastatic melanoma. At microscopy, the excised metastatic lymph nodes showed mostly epithelioid and spindled tumor cells and, in some portions, foci of chondrosarcomatous differentiation (Figure 3A). Immunohistochemistry (IHC) analysis performed on the sample obtained from the inguinal lymph node showed that tumor cells were positive for S‐100 and HMB‐45 (Figure 3B and C) and negative for MART‐1 and SOX‐10 (data not shown).

According to the AJCC 8th edition of TNM, the pathological stage of the tumor was IIIB (pT1aN2bM0).^9^

Molecular analysis was performed on both cutaneous melanoma and lymph node metastasis. Mutational analysis evidenced *BRAF* V600E variants on both primary tumor and metastasis (Figure 3D‐F).

At that time, patient came to our attention and adjuvant therapy with low‐dose interferon alpha (IFN‐α) was, firstly, administered, which was well‐tolerated. Later on, based on the molecular characteristics of the primary tumor (*BRAF* mutated), the patient was treated with a BRAF/MEK inhibitor combination therapy (dabrafenib 300 mg and trametinib 2 mg daily). The treatment was also well‐tolerated, without any relevant adverse effects.

After 3 months of therapy, FDG‐PET showed a substantial metabolic normalization of nodal metastasis, with only a weak and focal uptake at the caudal lymph node (SUVmax 2.7 vs 13). Four months later, after the patient had received a total of 7 months of therapy, FDG‐PET confirmed the positive metabolic response of the lymph nodes, without any evidence of distant metastasis (Figure 2B).

Patient continued dabrafenib/trametinib combination therapy, and at 20 months after nodal excision, there were no clinical or instrumental signs of disease recurrence. Considering the complete metabolic response after “neoadjuvant therapy” and the long‐lasting target therapy the patient underwent in the absence of disease recurrence, treatment with dabrafenib and trametinib was dismissed. At the next follow‐up visit (3 months after treatment ended), the patient was well and disease‐free.

## 3 DISCUSSION AND CONCLUSION

Among the histopathological patterns of melanoma, chondrosarcomatous is very rare.^3^ Few cases of pure chondrosarcomatous melanoma have been described in the literature.^10^, ^11^ To our knowledge, the most recent case describes the occurrence of chondrosarcomatous differentiation in a *lentigo maligna* of a 72‐year‐old woman's scalp.^12^ In this case, tumor analysis showed no evidence of *BRAF* mutation and the patient underwent rapid disease progression following primary tumor resection.

The mechanisms involved in the acquisition of different histological patterns and their clinical and prognostic significance are still unclear. It has been proposed that differentiation could represent a host response to injury.^3^ In fact, most of the reported cases of cartilaginous differentiation seem to have, as common feature, a previous traumatic event, which could drive fibroblasts to cartilaginous differentiation. The overexpression of MIA (melanoma‐inhibiting activity) factor, a soluble autocrine growth factor expressed in most metastatic melanomas, has been suggested to have a relevant role.^13^, ^14^, ^15^

Differential diagnoses of melanoma with divergent mesenchymal differentiation include soft tissue neoplasms with abundant extracellular matrix, such as myxoid liposarcoma, extra‐skeletal myxoid chondrosarcoma, chordoma, myxoid malignant fibrous histiocytoma, and malignant peripheral nerve sheet tumor.^16^

Immunohistochemistry can help in the differential diagnosis, even if there is a significant overlap in the staining patterns of these neoplasms. Moreover, in some cases, melanoma can show an abnormal immunophenotype (such as cytokeratins and smooth muscle actin immunoreactivity) or a total lack of expression of the most common markers of melanocytic differentiation, such as melan‐A and HMB‐45.^7^

It is not possible to draw conclusions about the prognostic significance of the histological variant described here because of the low number of reported cases, all of which show very different behaviors.^3^, ^8^ However, chondrosarcomatous differentiation may be present in the primary tumor or arise in the metastatic lesions, as in this case.^14^

Our case is an example of multidisciplinary management. This therapeutic approach is fundamental, especially in the setting of stage IV melanoma, where prospective clinical trials evaluating the most appropriate sequence and timing of systemic therapy and surgical resection are lacking.

Based on the presence of a *BRAF* mutation in the primary tumor, the patient was treated with a BRAF/MEK inhibitor combination therapy, which was well‐tolerated and led to an almost complete metabolic response, as expected.^17^ This made the patient a good candidate for metastasectomy, because she had a low and localized disease burden that was responsive to systemic treatment.^18^

We also report a “neoadjuvant,” premetastasectomy therapeutic approach in our patient with this rare histological variant of melanoma, with an almost complete metabolic response, as in the case of classic melanoma.

The additional peculiarity of this case was the persistence of the *BRAF* V600E mutation in the metastatic recurrence of melanoma, with chondrosarcomatous differentiation. To the best of our knowledge, this is the first report of such a case in the literature.

We conclude that the presence of a *BRAF* mutation may be a predictive factor of response to BRAF‐MEK inhibitor combination therapy in this rare variant of melanoma.

## 4 ETHICS STATEMENT

Patient was aware that data could be published.

## CONFLICT OF INTEREST

None declared.

## Data Availability

Not applicable.
